# The Assessment of Trihalomethanes Concentrations in Drinking Water of Hamadan and Tuyserkan Cities, Western Iran and Its Health Risk on the Exposed Population

**Published:** 2019-04-06

**Authors:** Azam Nadali, Alireza Rahmani, Ghorban Asgari, Mostafa Leili, Hosein Ali Norouzi, Afsaneh Naghibi

**Affiliations:** ^1^ Department of Environmental Health Engineering, Hamadan University of Medical Sciences, Hamadan, Iran; ^2^ Department of Environmental Health Engineering, Research Center for Health Sciences, School of Public Health, Hamadan University of Medical Sciences, Hamadan, Iran; ^3^ Department of Environmental Health Engineering, Vice-Chancellor in Health Affairs, Hamadan University of Medical Sciences, Hamadan, Iran

**Keywords:** Trihalomethanes, Health risk assessment, Drinking water, Iran

## Abstract

**Background:** The aim of the present study was to calculate and to assess the potential lifetime cancer risks for trihalomethanes from consuming chlorinated drinking water in Hamadan and Tuyserkan cities, western Iran in 2016-2017.

**Study design:** A cross-sectional study.

**Methods:** Seventy-two water samples were collected from the distribution systems and from the outlet of water treatment plants (WTPs) and the experiments were carried out to determine the desired parameters. All the sampling and measurement methods were according to Standard Methods. The obtained data were analyzed using SPSS software.

**Results:** The mean concentration of total THMs in the summer and winter was 42.75 and 17.75 μg/L, respectively, below the WHO and Iranian standard. The positive correlation was observed between temperature and THMs levels. Moreover, THMs concentration in Shahid Beheshti’s WTP was several times lower than in Ekbatan’s WTP. Chloroform, the dominant species of THMs, was identified at different sampling points. The highest cancer risk in Hamadan was 1.4×10^-5^ and 4.8×10-5 for male and female, respectively; and the cancer risk was obtained to be 5.6×10^-7^–2.26×10^-6^ in Tuyserkan.

**Conclusion:** The drinking water obtained from the studied area is safe in terms of THMs concentration. Nevertheless, the highest cancer risk was higher than the EPA’s acceptable level of 10^-6^ .

## Introduction

Today, the disinfection process has been identified as one of the essential steps in the water and wastewater treatment plants. Although various techniques and materials have been used for this purpose, chlorine has found as the most common and successful chemical^1^. Despite the various positive characteristics, the immense use of chlorine and its reactions with natural organic compounds (NOMs) existed in the surface water resources is led to formation of the disinfection by-products (DBPs), i.e., trihalomethanes (THMs), haloacetic acids (HAAs), haloacetonitriles (HCNs), haloketones (HKNs), and chloropicrin^2,3^.

Trihalomethanes including chloroform (CHCl_3_), bromodichloromethane (CHCl_2_Br), dibromochloromethane (CHClBr_2_), and bromoform (CHBr_3_) are the most predominant among them^4^. The formation of THMs in water’s distribution system is controlled/influenced by various parameters including pH, chlorine dose, water temperature, TOC content of the water, and resident time of water within the system^5^. The presence of some evidence towards their detrimental health effects such as spontaneous abortion, birth defects, prematurity, low birth weight, intrauterine growth retardation, and even cancers has increased environmental and human health concerns^6^. The exposure and uptake of THMs can happen through the drinking, contact with the skin or by inhalation^7^. THMs considered as systemic toxicants known to induce multiple organ damage, even at low levels of exposure. They are also classified as human carcinogens (known or probable)^8,9^. The maximum allowable level for these compounds has initially announced to be 100 μg/L as annual average; however, the U.S. Environmental Protection Agency (EPA) has established 80 μg/L as MCL in 1998 and, in next phase, it is expected to decrease its level to 40 μg/L as the maximum contaminant level goal (MCLG)^10, 11^.

Recently, several studies have reported the presence of THMs in the drinking water from various parts of Iran, such as Tehran^12^, Khuzestan^13^, Shiraz^14^, etc. the concentration of trihalomethanes was examined in the water distribution network of Shiraz city ^14^. THMs concentrations at all monitoring sites were lower than the standard value, and the highest concentration of THMs was related to the areas where the surface waters used as the source of water supply. The concentration of trihalomethanes compounds was investigated in the water distribution network of Ahwaz City ^13^. The concentration of THMs in summer was 1.6 times higher than its concentration in the spring and 1.4 times greater than its concentration in autumn. In Tehran, the concentration of chloroform investigated in drinking water and the concentration of chloroform in the distribution network was 3.3 times the outlet of the treatment plant^12^.

Considering the Hamadan and Tuyserkan cities, both surface and groundwater resources are used to supply drinking water, that such study has not been conducted, thus, the aims of this study were to assess the THMs concentration and the factors associated with their formation potential in drinking waters and to calculate the numeric value of cancer risk caused by exposure to chlorinated drinking water.

## Methods

### Study area

Hamadan City is the capital of Hamadan Province and is accounted for as one of the biggest cities in the west of Iran. This city is located between longitudes 48° 31′ E, and between latitudes 34° 48′ N, and its urban area are about 56 km^2^. This city has lied at an altitude of 1850 m above sea level and in the northern part of Mount Alvand. Based on the last census, this city has a population of 676,105. Hamadan has a favorable climate, with four distinct seasons. It has also the cold winters and the rainfalls of this city usually occur in the winter and spring. The annual average temperature and precipitation of this city are 11.3 °C, and 317.7 mm, respectively ^15^.

Tuyserkan is a city and capital of Tuyserkan County, Hamadan Province, Iran. The population of the city was 101,666 people. Both surface and groundwater resources are used to supply the water requirement of most residents of this township^16^.

Hamadan has two water treatment plant for supplying required drinking water, along with the wells, including Shahid Beheshti and Ekbatan WTPs. Shahid Beheshti WTP has a nominal capacity of 1000 L/s. The inlet flow to this treatment plant is supplied from two sources: a) Ekbatan, and b) Abshineh dams. The processes used in this treatment plant are as follows: pre-sedimentation, pre-ozonation, coagulation with alum and lime (to adjust the pH), clarification using two Pulsators^®^, rapid sand filters, and final chlorination. The nominal capacity of Ekbatan water treatment plant is up to 400 L/s. In this treatment plant, the primary disinfection process with chlorine is used to eliminate the pathogens and the factors causing the taste and odor. In the next step, after adding the disinfectant (poly aluminum chloride), the water enters the solids contact clarifier (Accelerator^®^). Finally, in the process of water filtration, the water is introduced into the rapid sand filters, and, after final chlorination, is entered the water storage tanks.

This descriptive cross-sectional study was conducted to determine the concentration of THMs in the distribution systems and at the water treatment plants of the Hamadan and Tuyserkan in 2016-2017. The samples were collected from the inlet and outlet of the water treatment plants. Moreover, to determine the concentration of THMs in the distribution systems, 6 points in the distribution system were selected and the sampling was performed in these points in both winter and summer seasons. To do this, seventy-two water samples were collected. The samples were collected at the points specified using 300 mL glass bottle at the points specified using 300 mL glass bottle. All the glassware was previously washed with phosphate detergent and then washed with distilled water and dried at 105 °C for 1 h. Furthermore, the residual chlorine was neutralized by adding the 1.5 mL of 10% sodium thiosulfate. After sampling, samples were transferred to the laboratory using the cool box at the temperature of ≈ 4 °C and, immediately, the extraction of the compounds from the samples was carried out.

### Chemicals and analytical methods

All the chemicals used were of analytical-reagent grade. Water temperature, pH, free and combined chlorine, and turbidity were measured at the sampling point concurrently with sample collection with thermometer, portable pH meter; colorimetric DPD test kit, and turbidimeter (Turbi Direct, Lovibond, Germany), respectively, according to the standard methods^17^. Simultaneously, other samples were taken from the mentioned points to measure other parameters, including THMs and TOC concentrations. TOC concentrations were measured using TOC analyzer, Vario, German Elementar Company.

For extraction of samples and to determine the concentration of THMs, the 6232B method mentioned in the Standard Methods^17^ was used. Extracting the samples was done using n-heptane solution as follows: 2 mL of water was mixed with 2 mL of heptane solvent in a capped vial and, after mixing of these two phases for 30 min, the water phase was discharged from the vial by syringe. The extracted samples were stored at 2 to 4 °C in the refrigerator. The standards for THM in heptane solution were prepared in a concentration range of 10 to 200 μg/L in the 2 mL capped vials. The temperature of the standard and sample vials was stabilized at 25 °C just before the analysis. A Gas Chromatography equipped with Mass Spectroscopy (GC-MS) (Agilent, 7890A, UK), was used for the determination of THMs levels (Table 1). Samples were injected into the headspace in a volume of 100 μL by a syringe, and THMs in each sample were kept according to the time, and the sub-peak area was identified and measured quantitatively and quantitatively^12^.

**Table 1:** GC-MS specification

**Gas Chromatography Specifications**	
Model	Agilent 7890A
Detector Specification
Type & Model	Mass 5975C
Detection limit	5µm
Column Specification
Type	Agilent HP-5MS
Length	30 m
diameter	0.25 mm
Film thickness	0.25 µm
Carrier gas specification
Type	Helium
Flow rate	1 mm/min
Purity	99.999
Injection volume	1000 µL

### Statistical analysis

All statistical analyses were performed using SPSS V. 16.0 (Chicago, IL, USA). To measure the mean differences of THM concentration in the two seasons, the Wilcoxon signed rank test was performed, and to compare the mean concentration of THMs in summer and winter with the WHO guideline, one-sample *t*-test was used. The correlation between THM concentrations and the other water quality parameters (e.g., Temperature, TOC, and etc.) were evaluated using the Spearman's rank correlation (*Ρ*).

### Exposure and risk assessment

Human health risk assessment was carried out for the chloroform measured in drinking water samples from Hamadan and Tuyserkan, according to the US Environmental Protection Agency (USEPA) guideline,^18^. The risk assessment was evaluated for various routes of THMs exposure, i.e. oral, dermal (skin contact) and inhalation. Showering, bathing and oral consumption activities of water were mostly categorized as dermal, inhalation and oral exposure. The chronic daily intake (CDI) and corresponding slope factor (SF) were applied to determine the unit cancer risks. The CDI for each exposure route, i.e. oral, dermal and inhalation was only calculated for chloroform because of its higher content in the drinking water compared to the other THMs (Eqs (1) to (3)^19^.

(1) $${\text{CDI}}_{\text{oral}}\text{(}mg/\text{kg}\cdot \text{day)=}\frac{\text{Cw}\times \text{IR}\times \text{EF}\times \text{ED}\times \text{CF}}{BW\times AT}$$

(2) $${\text{CDI}}_{\text{dermal}}\text{(}mg/\text{kg}\cdot \text{day}\text{)=}\frac{\text{Cw}\times SA\times \text{F}\times PC\times ET\times EF\times ED\times CF}{BW\times AT}$$

(3) $${\text{CDI}}_{inhalation}\text{(}mg/\text{kg}\cdot \text{day}\text{)=}\frac{{\text{C}}_{air}\times VR\times \text{AE}\times ET\times EF\times ED\times CF}{BW\times AT}$$

where, Cw is the chloroform concentration in the water sample (µg/L); IR represents the ingestion rate of drinking water (L/day); EF is the exposure frequency (day/year); ED indicates the exposure duration (years); SA shows the skin surface area of the human body (m^2^); PC is the speciﬁc dermal permeability (cm/h); ET shows the exposure time (h/day); Cair is the concentration of THM in the breathing air zone (mg/L); VR is the ventilation rate (m/h); BW is the body weight (kg) and AT is average lifetime (days). In addition to U.S. EPA values, certain assumptions were considered to accommodate local factors.

In this study, the average body weight of male and female were considered as 70 kg and 60 kg, respectively, as well as adult average exposure of 30 yr (70 yr), were adopted^19^. Moreover, the amount of water ingested (2.5 L/day) was considered on moderate consumption level on the basis of the study carried out in the similar region ^10^, and skin surface area of 2 m^2^ was used ^20^. The concentrations of CHCl_3_ in the air (C_air_ for CHCl_3_) were used ^21^.

Total cancer risk (TCR) for each location was achieved by the summation of the individual risk estimation of chloroform (CHCl_3_). TCR is expressed as the probability of excessive exposure to carcinogenic over a lifetime period and calculated using the following equation:

TCR= ∑_i_SF×CDI (4)

where TCR is the unit risk estimate of CHCl_3_ (unit less of probability), ∑_i_ is the exposure routes, SF is the slope factor values ^10,22,23^, and as mentioned, CDI is the chronically daily intake. Table 2 shows the slope factor for chloroform, linked with lifetime cancer risk to the exposed population.

**Table 2:** Carcinogenic slope factors used for chloroform risk assessment via different routes^23,24^

**Routes**	**Slope factor [(mg/kg-day)** ^-1^ **]**
Ingestion	6.10×10^-3^
Dermal	3.05×10^-2^
Inhalation	8.01×10^-2^

### Ethical considerations

The Ethics Committee of Hamadan University of Medical Sciences approved the study protocol and its questionnaire.

## Results

In order to investigate the effect of ambient temperature on the concentration of THMs, the sampling was carried out in both warm and cold seasons. Summary of statistics including negative ranks, positive ranks, and *P-*value for the Wilcoxon signed rank test was shown in Table 3. In different sampling points, there is a significant difference between the mean THM concentration in summer and winter, where the temperature and the residual chlorine were higher in the summer than in winter. The mean concentration of total THMs in the summer and winter were 42.75±30.76 and 17.75±30.76 μg/L, respectively. The results of descriptive statics for pH, temperature, and turbidity during summer and winter seasons were shown in Table 4.

**Table 3:** The results of Wilcoxon signed rank test for the THM during summer and winter seasons

**THMs**	**Negative Ranks**		**Positive Ranks**		**Test statistics** ^a^	
	**Mean rank**	**Sum of ranks**	**Mean rank**	**Sum of ranks**	**Z**	***P*** ** value**
(THM winter)-(THM summer)	4.50	36.00	0.00	0.00	‒2.52^b^	0.012

^a^ Wilcoxon Signed Ranks Test ^b^ Based on positive ranks

**Table 4:** The results of descriptive statics for the variables during summer and winter seasons

**Parameters/Statistics**	**Summer**				**Winter**			
	**Min.**	**Max**	**Mean**	**SD**	**Min.**	**Max.**	**Mean**	**SD**
pH	6.80	7.50	7.21	0.21	7.00	7.80	7.42	0.26
Temperature (ºC)	12.00	23.00	17.66	3.00	0.00	10.00	4.25	3.25
Turbidity (NTU)	0.06	3.70	1.05	1.43	0.14	13.80	2.26	4.23

**Figure 1:**
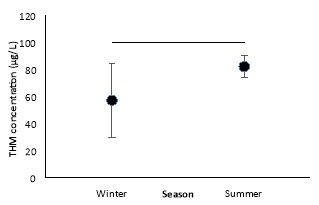
THMs concentrations in the summer and winter

The concentration of THMs both in summer and winter, were lower than that of the WHO’s guideline (100 μg/L) and Iran’s standard (200 μg/L) (Figure 1). Various parameters such as temperature, pH, free chlorine, turbidity, total organic carbon can affect the amount of THMs in water resources and distribution systems. To investigate the effect of these parameters, the Spearman correlation was performed. The main factors affecting the concentration of THMs in this study were residual chlorine, turbidity, and TOC. The statistical analysis with Spearman non-parametric correlation coefficients showed a positive correlation between residual chlorine and THMs concentration (R=0.86, *P*<0.05) and negative strong correlation (R>–0.8, *P*<0.05) between TOC concentrations, turbidity and THMs concentration, however, the correlation between pH and temperature with THMs concentration was not statistically significant (Tables 5 and 6).

**Table 5:** Spearman correlation coefficients between THMs concentrations and other parameters during the summer season

**THMs**	**pH**	**Cl**_2_	**Temperature**	**Turbidity**	**TOC**	**Chloroform**
R	‒0.40	0.86	0.80	‒1.00	‒0.80	0.80
*P* value	0.601	0.042	0.200	0.001	0.023	0.021

**Table 6:** Spearman correlation coefficients between chlorine and turbidity, between chlorine and TOC, with THMs in summer and winter seasons

**THM**	**Cl/Turbidity**	**Cl/Turbidity**_in_	**Cl/TOC**_in_	**Cl/TOC**
R	0.86	0.08	0.78	0.90
*P* value	0.001	0.767	0.001	0.002

The potential of THMs formation in Shahid Beheshti WTPs and the distribution systems supplied by this treatment plant was several times lower than that of Ekbatan WTPs. The mean concentration of THMs in Shahid Beheshti WTP and corresponding serviced distribution systems was in the range of 10 to 26 μg/L, while it was between the 10-120 μg/L for the Ekbatan WTP in both seasons. Accordingly, the highest and lowest concentrations were measured at the end of the distribution systems and at the inlet of the treatment plant. Chloroform, the dominant species of THMs, was identified at all sampling points. In Ekbatan water treatment plant and after the final chlorination, THMs concentrations greatly increases, as it was almost three times higher than the Shahid Beheshti WTP. Moreover, the concentration of THMs in Tuyserkan varied from 10 μg/L at the WTP outlet to 47.5 μg/L at the end of the distribution systems.

**Figure 2:**
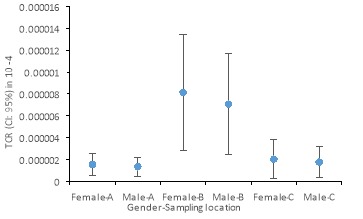
Average predicted risk upon study location and gender, A) Shahid Beheshti source, B) Ekbatan source, C) Tuyserkan source

Cancer risk assessment through ingestion, dermal and inhalation exposure was performed in the Hamadan and Tuyserkan distribution system. Results of 95^th^ percentile values of total cancer risk (TCR) are shown in Figure 2. Maximum unit cancer risk for CHCl_3_ was observed in the downstream of the distribution system for Ekbatan source, the downtown area.

**Figure 3:**
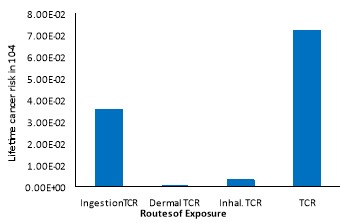
Lifetime cancer risks from chloroform via different routes

Figure 2 shows that the highest cancer risk is related to the end of the distribution network fed by the Ekbatan WTP. The TCR in the Shahid Beheshti WTP and the distribution network fed by this plant is 5.5×10^-7^–3.1×10^-6^. This value is 5.6×10^-7^–4.8×10^-5^ for the Ekbatan treatment plant and its serviced distribution network and 5.6×10^-7^–2.26×10^-5^ for the city of Tuyserkan. The ingestion route has the highest cancer risk and followed by the inhalation and dermal exposure, where the corresponding average risk for ingestion, dermal, and inhalation routes were 3.5×10^-6^, 4.5×10^-8^ and 3.3×10^-7^, respectively (Figure 3).

## Discussion

Figure 1 & Table 3 indicated that in both seasons, the concentration of THMs was lower than the WHO’s guideline^24^ and Iranian standard level^25^ which it is representative of a desirable and relatively safe level of THMs in drinking water. It was consistent with the results of other studies conducted in our country.

Our results revealed that THMs concentration in summer was higher than that of winter. These results were in agreement with the results of Uyak and Toroz (2005)^26^ study conducted to assess the effect of seasonal changes in the concentration of THMs in the Istanbul water distribution system. Concentrations of THMs in summer were 1.5 times more than the THMs concentrations in the spring. In another study, for all sampling sites, the concentration of THMs in the warm months (August) was significantly higher than that of the cold months (such as February)^27^. Increasing the temperature can accelerate both the decomposition and formation of THMs, therefore, one reason for increasing the THM concentration in the summer compared to the winter relates to the higher temperature in the summer. The concentration of THMs also increased by increasing the temperature but this relationship was not statistically significant (*P*-value>0.05). Increasing the concentration of THMs by increasing the temperature achieved at the temperatures beyond 30 °C, whilst the highest temperatures measured in the present study were 20 °C, therefore, it is not expected to have such significant change in final concentration of THMs. In this case, a study conducted to monitor the concentration of THMs in the Isfahan water distribution system and its zoning by GIS but did not show any significant relationship between the temperature and the formation of the THMs ^28^.

Increasing the length of the pipes in distribution system accompanied by increasing of the contact time between the chlorine and water’s content in the distribution systems fed from Ekbatan and Tuyserkan treatment plants, which in turn led to the increases of total THMs concentrations. Similar trends were also reported in other studies. For example, as the residence time of water in distribution system increases, the concentration of THMs was also increased significantly and is slowed down after 2 h ^29^. This can be due to decreasing the amount of residual free chlorine and reactive substances after this time. The total THMs (TTHMs) increases with increasing the residence time and it is reported that the formation of these compounds can be continued in drinking water until the disinfectant residuals and precursors are present ^30^.

pH is one of the most important factors affecting the THMs concentration in drinking water. The results of our study showed that although with increasing pH, the amount of THMs decreased, this difference was not significant (R=–0.4, *P*-value>0.05). THM concentrations as a function of pH variation were low because mean pH values of the samples changed in a low range (less than one unit). This finding is similar to another study ^28^, conducted on the concentration of THMs in the Isfahan water distribution systems, where it was not an increase in the concentration of THMs because of the low pH changes. The increase of THMs concentrations with the increases of pH could be due to this fact that the higher pH values are led to increasing the OH ions in the solution and developing the dissolution of THMs precursors, such as humic acid, and as a result, increases chloroform concentrations. This effect is seen in alkaline pH (8 or more). This inverse correlation may be due to the fact that the concentration of other components which increases the formation of trihalomethanes, such as organic precursors, is low^31^.

The concentration of chlorine and its residual in water is also an important factor in the formation of the THMs, in which the increase of chlorine concentration is led to an increase in the formation of the THMs, thus the reduction of chlorine concentration has been introduced as a means for decreasing the formation of the THMs^32^. Based on the Spearman correlation coefficients and the significance level that presented in Table 5, there is a direct correlation between the formation of THMs and chlorine residual concentrations, which are consistent with the results of other studies. For instance, the effect of the factors affecting the formation of the THMs investigated and observed that the amount of THMs formation increased by increasing chlorine levels ^33^.

As shown in Table 5 and based on Spearman correlation coefficients, a significant negative correlation is observed between the amount of TOC and the formation of the THMs. The results are similar to the findings in Isfahan^28^. The possible reasons for the negative correlation are as follows: all organic compounds do not necessarily result in the formation of disinfectant by-products, and TOC does not differentiate between various chemical compositions that consisting of the precursor compounds^28^.

Moreover, as seen in Table 5, there was a negative correlation between the amount of turbidity and the amount of THMs formation. The water turbidity in the studied treatment plants was not high enough after the treatment process, as it was reduced to below the 0.5 NTU as a standard, which this can be the reason for this negative correlation between the turbidity and the formation of THMs. Despite the high turbidity of raw water, turbidity removal processes in the treatment plant resulted in the increases of the removal of THMs precursors, which in turn led to lesser THMs production after chlorination ^34^.

Regarding that pre-ozonation has been used in Beheshti water treatment plant before the final chlorination step and given that ozone is a strong oxidation agent that reacts with many organic and mineral compounds in water, thus ozone can change the chemical profile of the natural organic matters (NOMs), hence affect the formation of chlorine and chloramine compounds^35^. Generally, the ozonation enhances the biological degradation of NOMs in water by converting larger organic molecules into smaller ones, which it tends to easily be decomposed. Ozonation can reduce the concentration of organic matter by 25-30%^35^. The concentration of THMs in the Shahid Beheshti WTP and the corresponding distribution systems was very low, which could be due to the use of ozonation as a pre-treatment step. Ozonation in neutral pH reduces the formation of THMs in the chlorination process^36^. Ozonation reduces THMs content by 35% and reduces THMs precursors by 42% ^35^.

The average of total cancer risk (TCR) in women was 8.2% higher than cancer risk in men. This can be due to body weight and dermal contact with chloroform. The cancer risk through the oral route accounts for 50% of the total risk. The lowest risk was related to the district serviced by Shahid Beheshti WTP, which can be due to the low concentration of chloroform. Pre-ozonation can reduce the THMs precursors that it also controls the formation of these compounds throughout the distribution network. The most cancer risk is related to the downstream of the distribution network fed by the Ekbatan treatment plant, which could be due to high concentrations of chloroform. The pre-chlorination and final chlorination process are used for disinfection on the water in this WTP. These processes increase the concentration of chlorine and increase the reaction between the THMs precursor and chlorine, hence increase the cancer risk in the population consumed treated water. Further, the average TCR from the ingestion route was 3.5 times greater than the EPA standard (10^-6^)^20^. The highest risk obtained from all three exposure routes is related to the downstream of the distribution network of Ekbatan (4.8×10^-5^), where it is a much higher risk than the EPA standard. In Tehran, the cancer risk of chloroform in the lifetime for surface water sources was 3.7×10^-5^, which can be in line with the results obtained in this study^21^.

The cancer risk was estimated for THMs through the several exposure routes and observed that cancer risk of ingestion route is much higher than the other two routes ^18^. Among the different THMs, the chloroform is the main THMs, which causes oral and dermal cancer risk. The results of this study indicate that the cancer risk related to chloroform is tens of times greater than the acceptable cancer risk provided by U.S. EPA which can be similar to our study.

In Ilam, the cancer risk of chloroform was 5.95×10^-6^‒6.59×10^ -6 10^. The average cancer risk in all sections was 6.26×10^-6^. A long-term review and meta-analysis study was performed towards the chloroform in drinking water and its relative health effects in Iran^37^. The average chloroform concentration (based on 14 studies) is 24 μg/L, which is lower than the EPA guidelines and the national standard. The authore used THQ (Target Hazard Quotient) to assess the non-carcinogenic risk. In all cases, except Mazandaran Province, individuals (men and women) in all age groups did not experience significant non-carcinogenic risk from chloroform in drinking water. The TCR in all considered cases was between 10^−6^ and 10^−5^ which means that these values were public health protective at low doses for the range of human variation^38^. The greatest part of the TCR was related to the oral ingestion route, followed by inhalation exposure, and lastly dermal absorption^38^.

## Conclusion

There was a correlation between residual chlorine, turbidity, and TOC with THMs concentration in the treatment plant and distribution systems. This correlation was weak in samples taken from the distribution systems fed by Shahid Beheshti treatment plant, which could be due to the use of pre-ozonation step in the treatment processes, which leads to decreasing the THMs precursors and, eventually, reducing the concentration of total THMs. Finally, the drinking water of the studied cities was safe and potable in term of THMs concentration.

The average of total cancer risk (TCR) in women was 8.2% higher than cancer risk in men. The lowest risk was related to the district serviced by Shahid Beheshti WTP, which can be due to the low concentration of chloroform. The most cancer risk was related to the downstream of the distribution network fed by the Ekbatan Treatment Plant, which could be due to high concentrations of chloroform. The highest risk obtained from all three exposure routes was related to this WTP of 4.8×10^-5^, where it was a much higher risk than the EPA standard.

## Acknowledgements

Authors would like to thank Deputy of Health and Hamadan's Water and Wastewater Company staffs, who contributed to the sampling and data collection.

## Conflict of interest statement

The authors declare they have no conflicts of interest.

## Funding

The research was funded by the Vice Chancellery for Research at the Hamadan University of Medical Sciences (Grant No. 9506093381).

## Highlights

- Chloroform was identified at different sampling points.
- The Measured THMs were well below the WHO and Iranian standard.
- The highest cancer risk is 1.4×10^-5^ for male in Hamadan.
- The highest cancer risk is 4.8×10^-5^ for female in Hamadan.
- The cancer risk is 5.6×10^-7^–2.26×10^-6^ in Tuyserkan.
